# Inexpensive High-Throughput Multiplexed Biomarker Detection Using Enzymatic Metallization with Cellphone-Based Computer Vision

**DOI:** 10.1021/acssensors.2c01429

**Published:** 2023-02-08

**Authors:** Neda Rafat, Lee Brewer, Nabojeet Das, Dhruti J. Trivedi, Balazs K. Kaszala, Aniruddh Sarkar

**Affiliations:** Wallace H. Coulter Department of Biomedical Engineering, Georgia Institute of Technology, Atlanta, Georgia 30332, United States

**Keywords:** diagnostics, multiplexing, point-of-care, COVID-19, computer vision

## Abstract

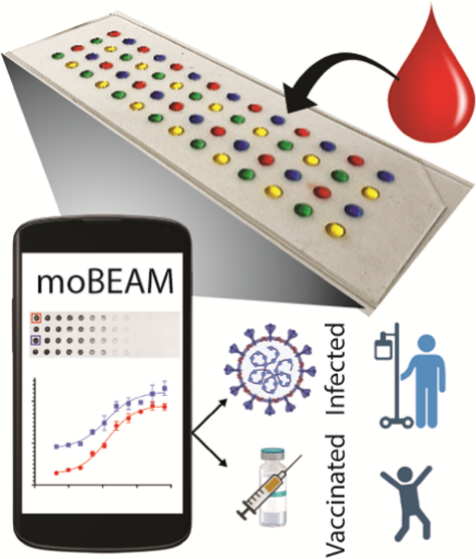

Multiplexed biomarker detection can play a critical role in reliable and comprehensive disease diagnosis and prediction of outcome. Enzyme-linked immunosorbent assay (ELISA) is the gold standard method for immunobinding-based biomarker detection. However, this is currently expensive, limited to centralized laboratories, and usually limited to the detection of a single biomarker at a time. We present a low-cost, smartphone-based portable biosensing platform for high-throughput, multiplexed, sensitive, and quantitative detection of biomarkers from single, low-volume drops (<1 μL) of clinical samples. Biomarker binding to spotted capture antigens is converted, via enzymatic metallization, to the localized surface deposition of amplified, dry-stable, silver metal spots whose darkness is proportional to biomarker concentration. A custom smartphone application is developed, which uses real-time computer vision to enable easy optical detection of the deposited metal spots and sensitive and reproducible quantification of the biomarkers. We demonstrate the use of this platform for high-throughput, multiplexed detection of multiple viral antigen-specific antibodies from convalescent COVID-19 patient serum as well as vaccine-elicited antibody responses from uninfected vaccine-recipient serum and show that distinct multiplexed antibody fingerprints are observed among them.

The COVID-19 pandemic has highlighted the importance of cost-effective point-of-care (POC) testing in controlling and mitigating infectious diseases.^1^ Scalable, high-volume testing is needed to prevent further spread and apply proper isolation, prevention of spread, and treatment strategies.^2^ As with tests for many infectious diseases, COVID-19 tests are divided into two main categories: diagnostic tests and serological tests.^3^ Molecular and antigen tests are the two leading types of diagnostic tests that can detect an active infection by measuring SARS-CoV-2-specific nucleic acids^4^ or protein antigens, respectively, whereas serological tests measure antibodies produced by the host immune system in response to SARS-CoV-2 infection.^5,6^

Serological tests are not effective for diagnosis of COVID-19 at early stages of infection. However, over time, viral antigen-specific antibodies are boosted in serum while the viral load decreases.^7^ This results in a higher accuracy for serological tests compared to molecular tests at middle to late stage of infection or for detecting prior infections.^8^ At the population level, serological tests can be used for large-scale seroprevalence studies to screen the immunity status of a community against COVID-19. Seroprevalence studies can provide a more accurate estimate of infections independent of disease symptoms.^9^ Serological tests can also provide information on the severity of infection by measuring antigen-specific antibodies^10^ and their functional profiles.^11^ Recently, we and others have shown that systems serology approaches, i.e., highly multiplexed comprehensive antibody profiling coupled to machine-learning-based analysis, can be used to predict mortality or survival outcomes in severe COVID-19.^12^ Additionally, heterogeneous individual vaccine efficacy and its durability can also be monitored via measurement of neutralizing antibody titers.^13^

Currently, commonly used COVID-19 serological tests include enzyme-linked immunosorbent assay (ELISA), chemiluminescence immunoassay (CLIA), immunofluorescence assay (IFA), and lateral flow assays (LFA).^14,15^ These methods work based on high binding affinity and specificity between viral antigens and host antibodies. ELISA and CLIA provide high-throughput and sensitive platforms for the detection of disease biomarkers.^15−17^ However, these methods require a relatively long detection time (2–8 h), trained technicians, and expensive and bulky plate readers for measuring the optical signals generated.^18^ Therefore, these techniques are limited to centralized laboratories and not practical for POC or resource-limited settings. Moreover, they are usually developed for the detection of a single biomarker and not suitable for multiplexed detection. Disease response often involves the interplay between many biological processes, and hence results in changes in multiple biomarkers rather than a single biomarker.^19,20^ Therefore, reliable and cost-effective multiplexed assays are essential to improve the diagnostic accuracy of many diseases.^21,22^ There are newer commercial ELISAs or bead-based sandwich assay methods for multiplexed immunoassays, but they are even more expensive and complex compared to conventional ELISAs.^23^ LFAs, developed based on the principle of sandwich immunoassays, are commonly used for POC testing due to their simplicity, speed, and low cost.^24−27^ But they usually offer only qualitative or semiquantitative results. They are also usually designed for single biomarker detection for individual tests and offer low to moderate sensitivity and limited flexibility in assay design.^28^

Over the past decade, research on the development of smartphone-based diagnostics has gained attention. With the continuous increase in the processing power as well as quality and quantity of built-in sensors, there is increasing interest in using smartphones in biomedical research and in the clinic. In particular, the last decade has seen an enormous improvement in the quality of smartphone cameras^29−32^ and a concomitant rise in their use as optical sensors.^30^ Often, when used as an analytical sensor, the smartphone camera takes the place of a traditional spectrophotometer. Less common, however, is leveraging advances in computer vision to analyze images taken by a smartphone camera. With proper attachments, hardware, and cellphone applications, they can be adapted as portable, versatile, and cost-effective read-out platforms for POC diagnostics^31,33^ and thus help to decentralize and democratize clinical laboratory tests.

Here, we present a low-cost smartphone-based portable biosensing platform for high-throughput, multiplexed, sensitive, and quantitative detection of biomarkers from small volumes (<1 μL) of serum samples. We developed an ELISA detection strategy based on enzymatic silver metallization^34^ that converts biomarker concentration to the localized surface deposition of an amplified, optically opaque, dry-state stable silver metal layer. This is then coupled with a smartphone-based computer vision application that enables an easy-to-use yet sensitive and quantitative optical readout. We term this platform as the **M**ultiplexed **O**ptical **B**ioassay using **E**nzym**a**tic **M**etallization or **MO-BEAM**. We demonstrate here the use of MO-BEAM for the high-throughput, multiplexed detection of SARS-CoV-2 viral antigen-specific antibodies from convalescent patient sera and monitoring of vaccine-elicited antibody responses.

## Materials and Methods

### Materials

Sodium hydroxide (S5881), poly-l-lysine solution (P8920), and bovine serum albumin (A7030) were obtained from Sigma-Aldrich. Reagent alcohol (BDH1156) and Tween 20 (97062-332) were obtained from VWR. Deionized water (DIW, LC267405), and phosphate buffer saline (PBS, 21-040-CVR) were obtained from Fisher Scientific. SARS-CoV-2 Spike recombinant protein (IT-002-032p) was obtained from Immunetech. SARS-CoV-2 (2019-nCoV) nucleocapsid-His recombinant protein (40588-V07E) was obtained from SinoBiological. Recombinant Protein A/G (6502-1) was obtained from BioVision. Biotinylated Bovine Serum Albumin (29130), HRP-Conjugated Streptavidin (N100), TMB substrate (34029), and 96-well microplates (15041) were obtained from Thermo Fisher. Mouse Anti-Human IgM-HRP (9020-05) and Mouse Anti-Human IgG Fc-HRP (9040-05) were obtained from Southern Biotech. EnzMet for General Research Applications (6010-45ML) was obtained from Cedarlane.

### Clinical Samples

Clinical serum samples were obtained from Ray Biotech, Inc. and Innovative Research, Inc. Consent forms were obtained from all sample donors prior to collection.

### PLL Coating

Standard microscope glass slides (25 mm × 75 mm) were cleaned with 10% NaOH/60% reagent alcohol in DIW for 2 h followed by rinsing with DIW thoroughly. Next, they were dipped in 30% PLL in 30 mM PBS for 30 min, rinsed with DIW, and spin-dried. PLL-coated glass slides were stored under vacuum in a desiccator at room temperature.

### PDMS Preparation

**Figure 1:**
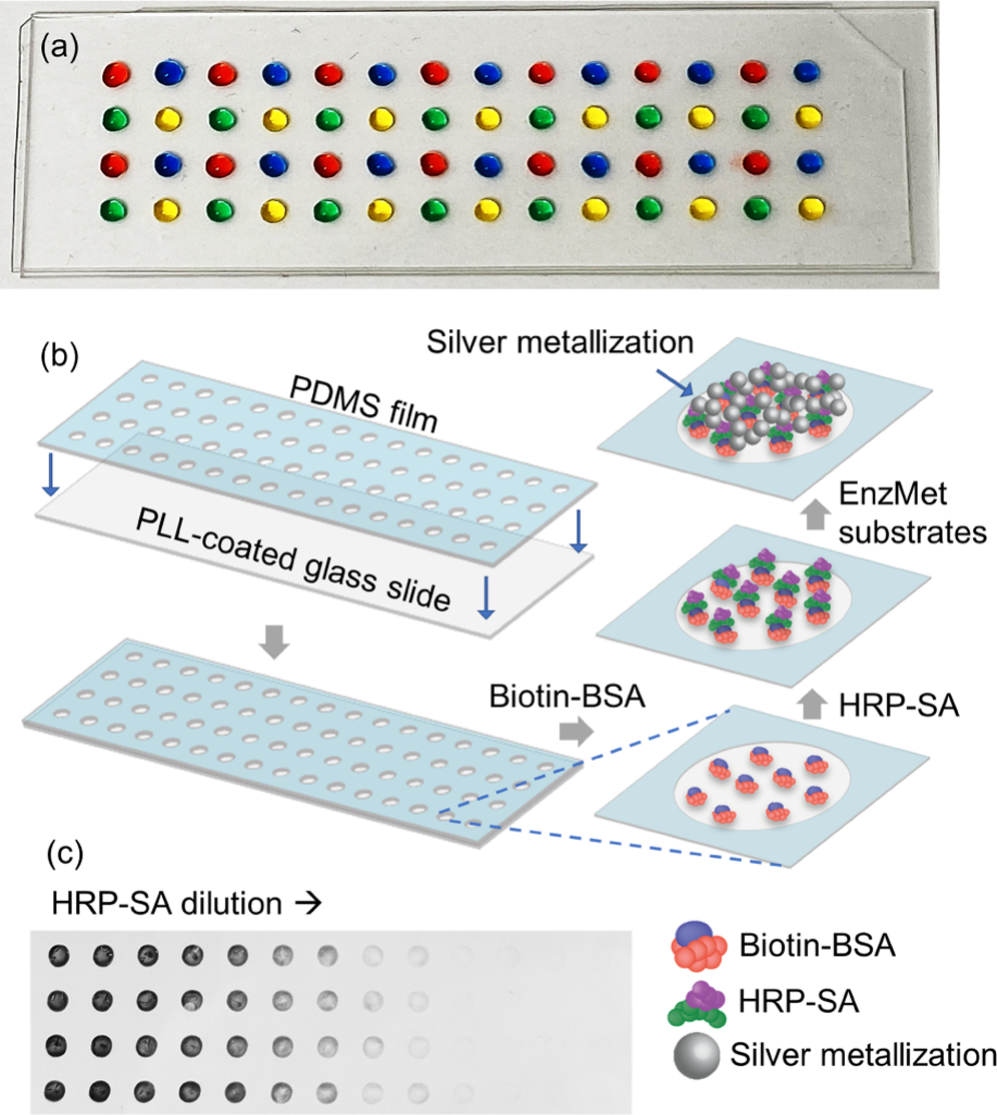
(a) Custom microwell array created using laser-cut PDMS film on PLL-coated standard microscope glass slide. (b) Workflow of enzymatically amplified silver metallization. Biotin-BSA was immobilized on each well, and after a blocking step, a range of HRP-SA concentrations were added to the wells. Then, enzymatic metallization substrates were added to deposit HRP-catalyzed silver metallization. (c) Deposited silver metallization on glass slide. Higher concentrations of HRP-SA generated denser and darker spots.

A thin polydimethylsiloxane (PDMS) film (0.1 mm thickness, Greene Rubber) was laser-cut to create an array of 2 mm wells. This was soaked in 5% Alconox in DIW for 30 min followed by rinsing with DI water. After air drying, scotch tape was used to remove any dust or particles before visually aligning and reversibly sealing with slides (Figure 1a).

### Biotin-BSA HRP-SA Model Assay

1 mg/mL biotin-BSA in PBS was added to each well and incubated for 1 h. All incubations were performed in a humidified chamber at room temperature unless otherwise specified. Next, the glass slide was blocked with 1% BSA in 0.1% Tween 20 in PBS (0.1% PBST) for 30 min and washed with 0.1% PBST and PBS. All washing steps were performed by placing the glass slide in a Petri dish filled with washing buffer on a plate shaker for 10 min. Then, the slide was dipped in DI water and spin-dried. Probe solutions, consisting of different HRP-SA dilutions were prepared in 1.5 mg/mL BSA in 0.05% Tween 20 in PBS (0.05% PBST). Next, probes were added to each well and incubated for 1 h. After two washes with 0.1% PBST and one wash with PBS, the slide was dipped in DIW and centrifuge-dried (Figure 1b).

### Silver Metallization

Equal volumes of enzymatic metallization substrate components A, B, and C were sequentially added and incubated for 4, 4, and 8 min, respectively. To stop the reaction, slides were dipped in DIW and dried.

### Immunoassay of COVID-19 Antigen-Specific Antibodies

COVID-19 antigens (S, N) or control proteins (BSA, Protein A/G) were prepared at 50 μg/mL in PBS and added to each well and incubated overnight at 4 °C. Next, the slide was blocked, washed, and dried as above. Antigen-modified slides were routinely stored at 4 °C for up to 2 weeks before use without any noticeable degradation of measured signal (Figure S1). Serum samples were diluted in 1 mg/mL BSA in 0.05% PBST, and 3 μL each of diluted samples was added to the wells and incubated for 1 h, followed by washing and drying again. A probe solution consisting of [1:400] HRP-anti-human IgG or HRP-anti-human IgM in 1.5 mg/mL BSA in 0.05% PBST was prepared, and this mixture was added to each well. After 1 h incubation, the slide was washed and dried, and the silver metallization step was completed as above (Figure 3a). Pooled COVID+ samples from 10 different individuals were first tested (Figure 3) followed by individual serum samples (Figure 5).

### Cellphone Application Development

The app was developed in Kotlin and Java using Android Studio. In addition to OpenCV for computer vision, several core Java and Kotlin libraries were used as well. The AAcharmodel library was used to display graphs within the app, and the Multik library was used for the implementation of a multidimensional array. The complete code for the app is available for download and use on Github at: https://github.com/MNBEL/MOBEAM

### ELISA

Microtiter plates were coated overnight at 4 °C with 50 μL/well of 2 μg/mL antigens in PBS. The plates were washed three times with PBS and blocked with 1% BSA in 0.05% PBST for 1 h at room temperature (RT) followed by three washes with PBS. Samples in 0.1% BSA in 0.05% PBST were added at 50 μL/well. After 1 h of incubation at RT, wells were washed three times with 0.05% PBST. Mouse Anti-Human IgG Fc-HRP at a dilution of 1:1500 in 0.05% PBST was added at 50 μL/well and incubated for 1 h at RT. All incubations were done on a shaker at 60 RPM. Following four washes with 0.05% PBST, 50 μL of TMB substrate was added. The reaction was stopped, after 15 min, by adding 50 μL of 1 M sulfuric acid. Absorbance was read at 450 nm using a microplate reader.

## Results and Discussion

### Enzymatically Amplified Silver Metallization in an Inexpensive Custom Microwell Array

To enable inexpensive and rapid high-throughput testing from small volumes of clinical samples, a custom microwell array was created (Figure 1a). Briefly, microwells were laser-cut in a thin PDMS film. This was then reversibly sealed to a standard microscope glass slide. This enables handling a large number (>50) of small samples and reagent volumes (<3 μL) in a leak-free manner during further use. For example, the image shown in Figure 1a was taken 1 h after the addition of the colored solutions, which showed no visible leakage. The microwell array was designed to enable dispensing using standard multichannel pipettes. Additionally, the shallow wells facilitate rapid slide-scale liquid handling steps as well as washing by dipping the whole glass side in wash buffer and spin-drying. Further, the PDMS microwell layer is easily peeled off as well to replace or reconfigure it.

Initially, binding assays of biotin-conjugated bovine serum albumin (biotin-BSA) with horseradish peroxidase-conjugated streptavidin (HRP-SA) were performed (see the Materials and Methods section) to study the enzymatically amplified silver metallization as shown in Figure 1b. Dark spots of silver metallization were observed on the surface of glass (Figure 1c) after washing and drying. Visually, the silver spot darkness was observed to be related to HRP-SA concentration with increasing darkness at increasing concentrations. This established both the dry-stable nature of the deposited silver and its ability to generate a visually readable darkness output.

### Cellphone-Based Computer Vision for the Detection and Quantification of Silver Metallization

To generate a quantitative readout of the assay without the use of any additional hardware, an Android-based cellphone app was developed. This automatically detects the silver spots from an image of the slide captured using the phone camera, quantifies the darkness of the silver metallization, and stores and plots the resulting data. OpenCV, an open-source library of real-time computer vision programming functions was used to build the object recognition and quantification aspects of this application.

**Figure 2:**
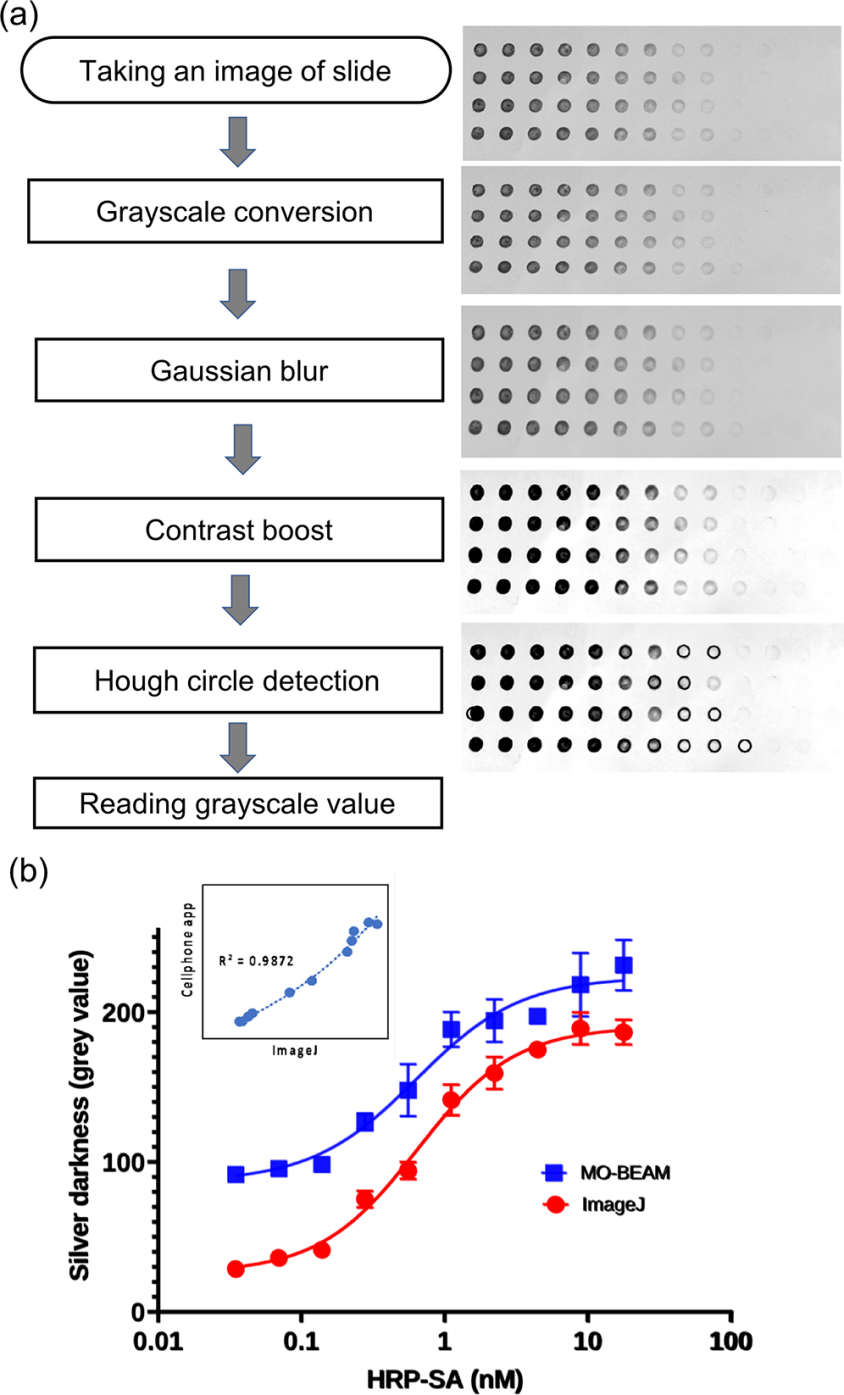
Quantification of silver metallization using the cellphone app. (a) Image preprocessing steps for quantification of silver darkness and their results. Key parameters at each step are also shown. (b) HRP-SA dilution curves obtained by the cellphone app and by ImageJ. Inset shows the high correlation of the two analyses.

Figure 2a shows the steps performed by the app and their results. A number of image preprocessing steps were performed. The input image was converted to grayscale, giving each pixel a single integer value between 0 and 255 that represents how dark it is, with 0 being perfectly black. Next, to reduce noise in the image, it was filtered using the *GaussianBlur* function in OpenCV.^35^ This replaces each pixel value with a weighted average of its neighboring pixels where the kernel, or shape of function used for averaging, is a Gaussian function whose standard deviation is selected as a blur parameter, σ. Finally, the image contrast was increased to make objects in the image easier to detect. This was done using the OpenCV *addweighted* function. As used here, this function multiplies each pixel in an image by a weight α then subtracts a scalar γ to adjust the brightness. Each pixel value in the new image was thus given by *f*′(*x*, *y*) = α* *f*(*x*, *y*) – γ where the α value is reported as the contrast parameter here. After preprocessing, the circular regions of the image were identified using the OpenCV function *HoughCircles*.^36^ Here, a radius, ρ, was defined as a parameter setting the region over which the average grayscale value was calculated. The pixels inside these regions have their grayscale values averaged together. The result was then classified based on the location of the region within the image. Replicates of each dilution were averaged, and the results were graphed using the MPAndroidChart library.^37^ After subtracting from 255 to invert the final readout to one that numerically increases with spot darkness, a readout that increased with analyte concentration was obtained as shown in Figure 2b (blue line).

Finally, the results obtained were also saved to a .csv file for further offline analysis and visualization as needed. To verify the results obtained from the cellphone app, ImageJ software on a desktop computer was also used to manually locate the spots and quantify the silver spot darkness values from the same image (red line). While the absolute values of the readout differ due to minor differences in manual placement of the quantification boundary, the limits of detection (LODs), as indicated by the inflection points of the curves, are seen to be similar for both techniques. To calculate the quantitative LOD, the standard deviation of PBS control was multiplied by 3 and divided by the slope of the calibration curve.^38^ Both techniques resulted in a similar LOD (∼100 pM), and the readouts obtained were found to be highly correlated (inset in Figure 2b). Figure 2b shows the results from a cellphone app plotted versus those obtained using ImageJ. A high correlation (*R*^2^ ∼ 0.99) is observed between the two measurements. Additionally, intra-assay coefficient of variation (CV), i.e., measure of variance between technical replicates run on the same glass slide, was found to be less than 5% and inter-assay CV, i.e., measure of variance between technical replicates run on different glass slides was found to be less than 8%. This is in an acceptable range compared to commercial immunoassays run on traditional equipment such as microtiter plates and plate readers.

### Detection and Quantification of COVID-19 Antigen-Specific Antibodies

**Figure 3:**
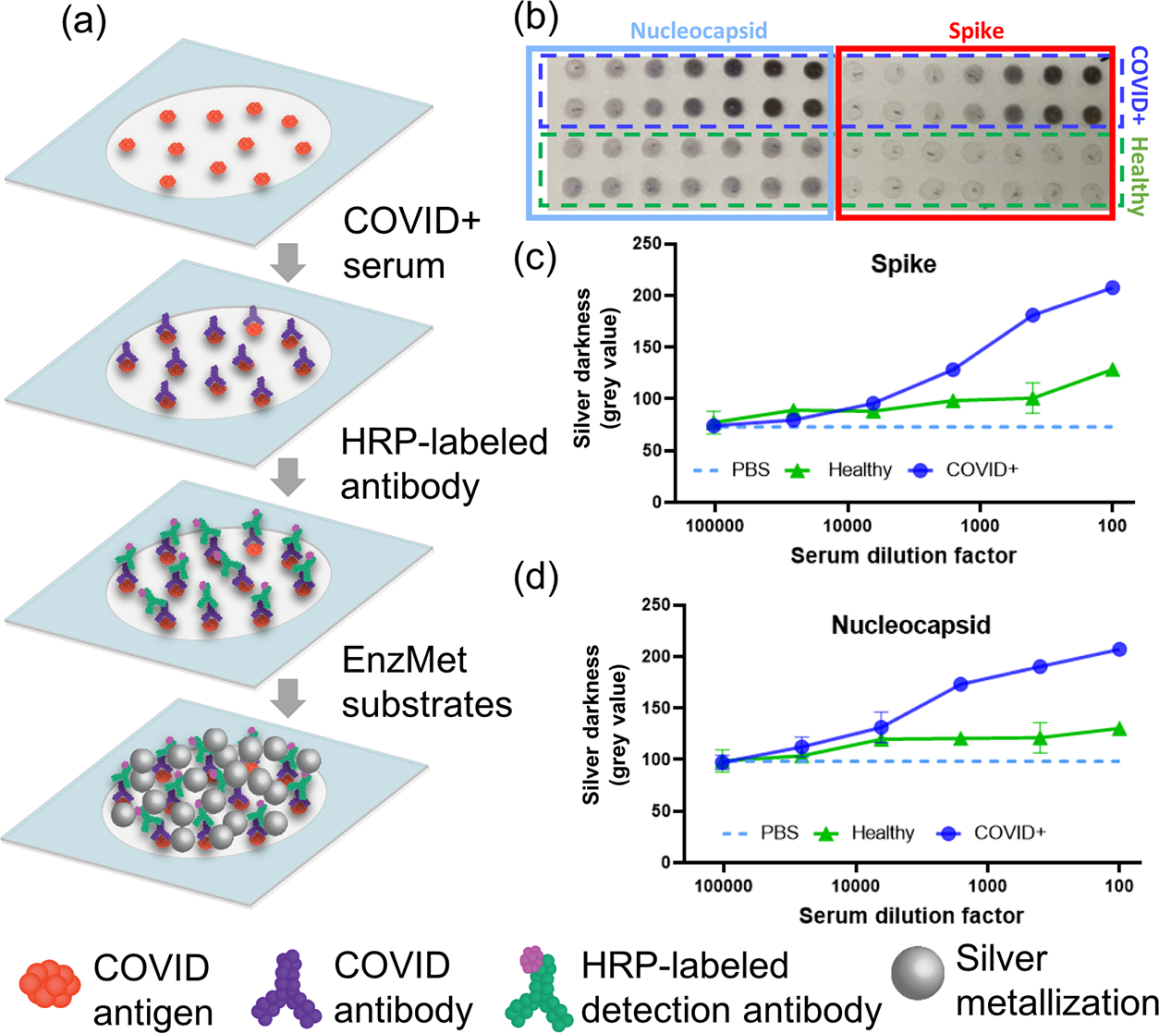
Detection of SARS-CoV-2 antigen-specific antibodies. (a) Immunoassay steps for the detection of SARS-CoV-2 antigen-specific antibodies via silver metallization. (b) Silver metallization deposited in response to the dilution of COVID-19 pooled serum sample, healthy pooled serum sample, and PBS control with anti-IgG probe. Quantification of silver darkness via the cellphone app for detection of human IgG against Spike (c) or nucleocapsid antigens (d).

Next, we set out to apply this platform for the quantitative detection of disease biomarkers such as viral antigen-specific antibodies in COVID-19. Human IgG/IgM antibodies against SARS-CoV-2 spike (S) and nucleocapsid (N) antigens (anti-S/N IgG/IgM) were selected as the target biomarkers. Immunoassays of IgG and IgM antibodies against S and N proteins were performed (see the Materials and Methods section and Figure 3a) using pooled serum from convalescent COVID-19 patients (*n* = 10) and pre-pandemic healthy serum as well as buffer (1XPBS) control. Total serum volumes less than 1 μL were used for each sample. Clear differences were observed in silver darkness between patients and healthy serum and the buffer control (Figure 3b).

This difference in signal between patients and healthy serum also indicates the specificity of the assay in being able to detect SARS-CoV-2 antigen-specific antibodies from a serum background. Note that healthy serum does show a small finite level of silver metallization, especially at low serum dilutions, i.e., high concentrations. This can be attributed to both preexisting cross-reactive antibody responses to endemic human coronaviruses as we have described in our recent work on antibody profiling in COVID-19^12^ as well as to nonspecific binding. Buffer control (1XPBS) showed little to no metallization, establishing lack of nonspecific binding of the HRP-labeled probe. Additional tests of assay specificity performed using antibodies against an irrelevant non-SARS-CoV-2 antigen as well as with a non-target-specific probe are shown in Figure S2. Results of the assays also showed that silver metallization density on glass increased with increasing concentration of serum, which results in a darker silver deposition. Dilution-dependent silver darkness can be observed for both S and N antigens for both IgG and IgM responses (Figures 3b and S3). Stronger silver response was observed for IgG compared to IgM response for this sample (Figure S3), potentially indicating sample collection at a later stage of infection.

Next, to obtain a quantitative readout, the app developed above was updated to classify each result based on the location of the region within the image. For example, if the user indicated that wells in the top right corner of the slide used S antigens and COVID-19 patient serum (Figure 3b), it was classified as such and plotted as a separate data series in the graph, as seen in Figure 3c,d. As described above, blank sample dilution buffer (1XPBS) and healthy samples are used as the negative controls.

Both the anti-S and anti-N immunoassays showed clear signals above controls and dilution curves, with a limit of detection (LOD) of 1:60,000 (1.7 pM) serum dilution for anti-S IgG and 1:70,000 serum dilution for anti-N IgG (1.5 pM). Note that the concentration of the antibodies in the clinical sample was defined here using a calibration curve based on a standard ELISA, run using the same clinical sample and a human anti-S IgG monoclonal antibody of known concentration (Figures S4 and S5). Thus, this platform successfully detected and quantified anti-S/N IgG/IgM biomarkers from serum sample volumes lower than 1 μL at an ∼pM scale sensitivity.

### Optimization of the Image Processing Parameters

**Figure 4:**
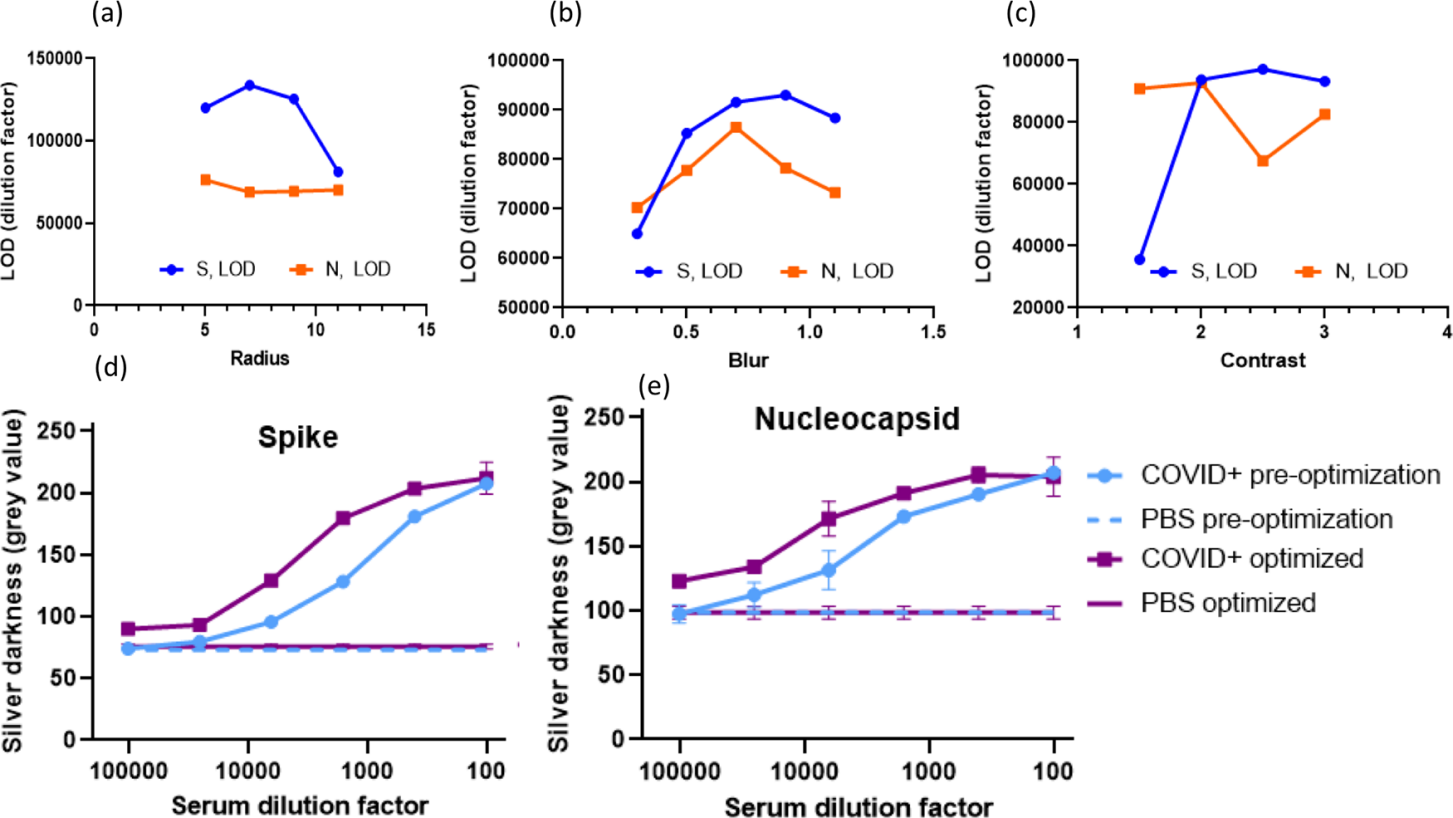
Optimization of parameters involved in preprocessing stages for cellphone app. (a) Effect of contrast on LOD. (b) Effect of blur on LOD. (c) Effect of radius on LOD. (d) Dilution curve for detection of spike human IgG before and after optimization. (e) Dilution curve for the detection of nucleocapsid human IgG before and after optimization.

We next varied and optimized the parameters used in the image processing steps in the cellphone app. As discussed earlier, a blur (σ) and an increase in contrast (α) were applied to the image to aid in object detection, and a radius of the microwell region (ρ) was used in which the darkness was quantified. A range of values for the radius, contrast, and blur parameters were tested to determine which parameters would result in the maximum LOD (Figure 4a–c). Decreasing the radius of the region over which the average grayscale value was calculated was found to increase LOD, with a size of five pixels giving the best results (Figure 4a). We hypothesize that this is because even wells that show overall low metallization exhibit a “coffee-ring” effect,^39^ where a ring of dark metallization forms on the edge of the well. Thus, excluding the edge of the circular region in which the microwell was detected improved LOD. The effect of blur on LOD followed a parabolic pattern, with a blur of 0.7 being the optimal value (Figure 4b). Plausibly, this is because some amount of blurring removes high-frequency noise such as small flecks and spots of metallization, while too high of a blur makes features undetectable. Varying contrast did not have a clear effect (Figure 4c). Optimized dilution curves for the detection of COVID-19 IgG antibodies against S and N antigens are shown in Figure 4d,e. Parameter optimization improved the separation between COVID+ and PBS control curves and thus improved the LOD from a dilution factor of 1:60,000 (1.7 pM) to 1:95,000 (1 pM) for anti-S IgG and from a dilution factor of 1:70,000 (1.5 pM) to 1:93,000 (1 pM) for anti-N IgG. Application of the optimized app parameters to the model assay results shown in Figure 2 is also included in Figure S6.

### Multiplexed Biomarker Detection from a Single Sample Droplet

**Figure 5:**
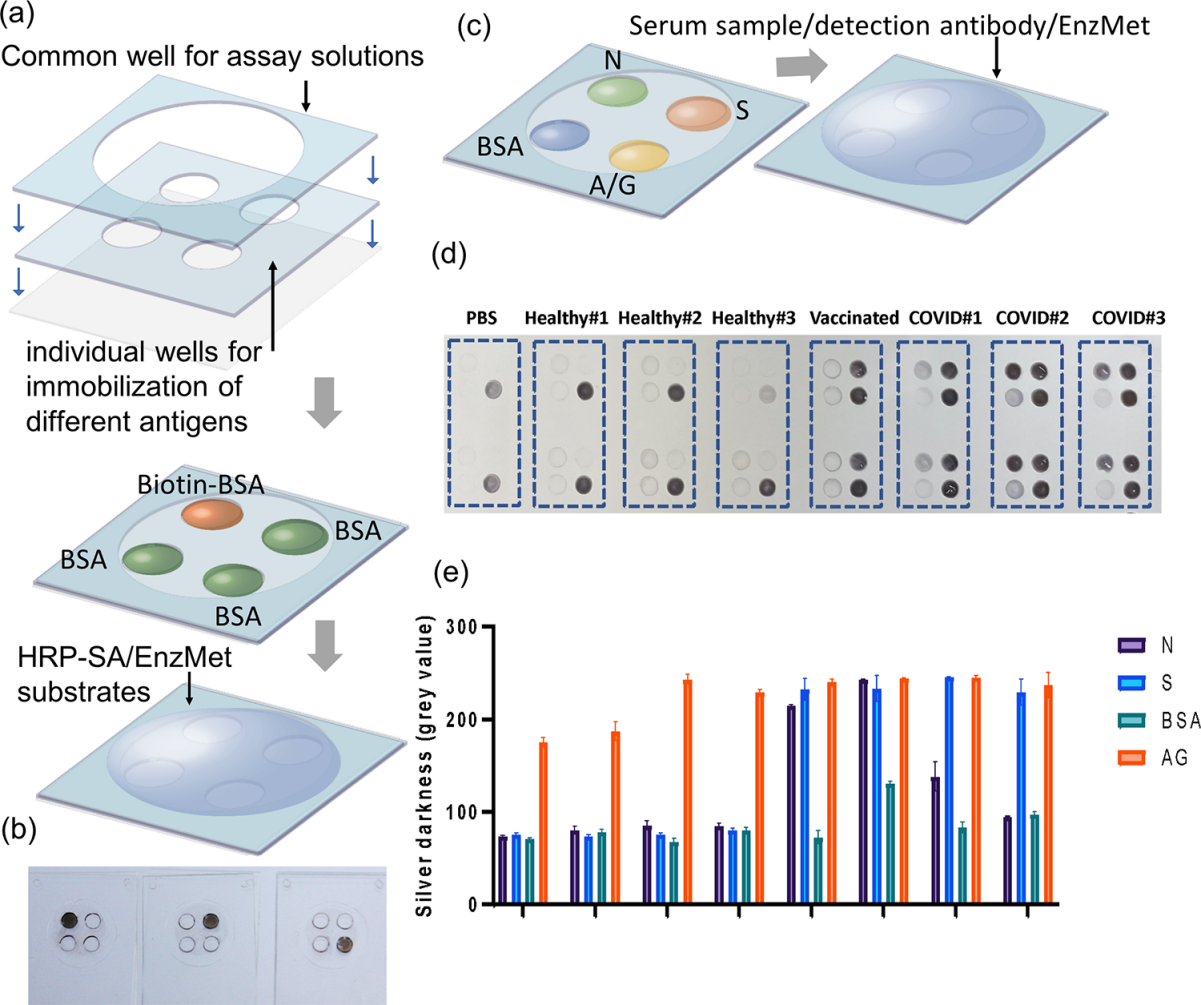
Multiplexed detection of biomarkers. (a) Two layers of laser-cut PDMS were used to create an array of four wells for immobilization of different antigens and a bigger well for sharing assay solutions between the antigen wells. (b) Demonstration of localized silver metallization using Biotin-SA chemistry. (c) Multiplexed detection of human IgG against S and N antigens. BSA and Protein A/G were used as negative and positive controls respectively. (d) Distinct multiplexed silver deposition patterns of buffer control, healthy controls (*n* = 3), S mRNA vaccine-recipient (*n* = 1), and COVID+ sera (*n* = 3). (e) Quantified multiplexed antibody responses buffer control, healthy control, S mRNA vaccine-recipient, and COVID+ sera.

Next, we tested the use of this platform for multiplexed detection of several biomarkers from a single small volume sample drop. To do so, two layers of PDMS were laser-cut and set up on the glass slide as earlier. The first antigen layer included four smaller (diameter: 1.5 mm) microwells for the immobilization of different capture antigens. The second sample layer included a larger (diameter: 6 mm) microwell covering all of the smaller wells for sharing a single drop of sample and other assay reagents between the smaller wells (Figure 5a). This multiplexing scheme was first tested using the model biotin-BSA and HRP-SA assay as above. Biotin-BSA was immobilized as the positive control on only one of the smaller wells while the remaining wells were coated with BSA as the negative control (Figure 5a). Then, HRP-SA was added to the larger microwell and the assay was completed as earlier. Silver metallization was observed only on positive control wells, and no metallization was found on negative control wells (Figure 5b). This shows that the silver metallization occurs locally, remains stable where it is formed, and does not diffuse away or cross-deposit to other wells even when the sample and probe are shared as a single droplet. Notably, this is unlike a conventional ELISA where the molecules causing the optical signal can diffuse in a solution. This enables multiplexed detection of biomarkers from a single sample drop without any concern for crosstalk of signals from adjacent spots with different capture antigens.

A multiplexed assay was then designed (Figure 5c) for the simultaneous detection of anti-S and anti-N IgG antibodies from a single drop of serum. Using the four-well antigen layer as above, S and N proteins, BSA (-ve control), and recombinant Protein A/G (+ve control) were immobilized in separate microwells. A single drop of serum was then added to the common sample well and the assay was performed as earlier. Individual convalescent COVID-19 patient (*n* = 3), uninfected vaccine-recipient (Pfizer mRNA, *n* = 1), and pre-pandemic healthy donor serum (*n* = 3) and buffer (1XPBS) control samples were tested in duplicate. Results of this assay showed the unique antibody signatures of each sample type (Figure 5d,e) that differentiated the sample classes from each other. Buffer (1XPBS) control samples and pre-pandemic healthy serum show no response except for the Protein A/G +ve control, which is expected to bind all (i.e., non-SARS-CoV-2-specific) human IgG from serum as well as the HRP-labeled mouse IgG used as probe here. Thus, a nonzero signal is obtained with the Protein A/G for the buffer, due to mouse IgG probe binding, and healthy controls due to human IgG binding. COVID+ serum was found to have both anti-S and anti-N IgG, although their relative concentrations varied across patients. Additional individual COVID+ (*n* = 10), healthy (*n* = 10), and buffer control samples were tested using the multiplexed assay and were found to have similar antibody signatures as above (Figure S7). This matches our and others’ earlier findings that SARS-CoV-2 infection results in antibodies against a larger set of viral antigens including S and N.^12,40−42^ Finally uninfected vaccine-recipient serum showed only anti-S IgG but no anti-N IgG, which is as expected since the vaccine used (Pfizer mRNA vaccine) contains only S mRNA and results in an immune response directed against the S antigen.^43^ This indicates the utility of this MO-BEAM platform in rapid and inexpensive vaccine response monitoring as well.

### Comparison with ELISA and LFA

**Figure 6:**
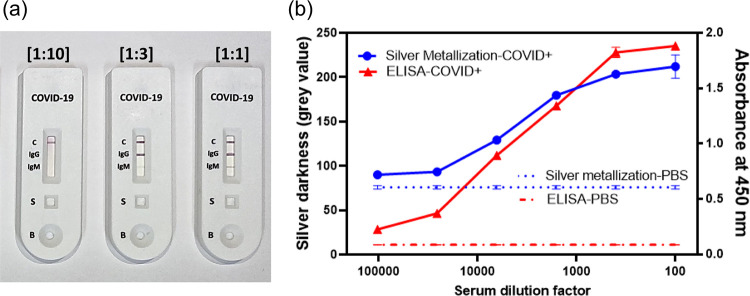
Comparison of the biosensor’s performance with optical ELISA (a) and LFAs (b).

Neither LFAs nor ELISAs are inherently multiplexable like the MO-BEAM platform developed here. However, we were interested to compare the other key performance parameters with a commercial LFA test and a conventional ELISA. Both LFA and ELISA were performed using the same COVID+ serum samples as tested with MO-BEAM earlier. LFA was only able to detect anti-IgG S from serum samples up to 1:3 dilution or less (Figure 6a). Note that three replicates of the LFAs were performed at each dilution and found to have identical results. Thus, the detection sensitivity of our platform was found to be 30,000× better than LFA while providing multiplexed and quantitative data from serum samples as low as 0.1 μL before dilution. Better sensitivity of detection is crucial for detecting antibody biomarkers post-infection whose titer drops over time. Therefore, better sensitivity and multiplexing in the MO-BEAM platform provide an important advantage over LFAs for screening disease biomarkers.

ELISA for anti-S IgG detection was performed on a 96-well microtiter plate using standard protocols (see the Methodssection) and read out using a plate reader. It resulted in a LOD of 1:400,000 dilution (0.25 pM) meaning approximately 4-fold better sensitivity than the 1:100,000 (1 pM) obtained with MO-BEAM in the present work (Figure 6b). While the current picomolar sensitivity of MO-BEAM was enough for detecting the antibody-based biomarkers tested here, the 4× gap vs ELISA represents an opportunity for further improvement in sensitivity. A number of other parameters, especially those related to surface capture density and kinetics of biomolecule binding can be explored to achieve this. Specifically, the charge-based antigen attachment method to PLL-coated glass may be improved upon, in terms of antigen density and orientation, via other methods such as covalent attachment and gel-like coatings. Further, the enzymatic metallization reaction itself is unexplored here for optimization as its detailed mechanism remains unknown. We have recently hypothesized^42^ that the silver deposition is self-limiting as the HRP that catalyzes the silver reduction itself serves as the nucleation site for sliver deposition. This eventually blocks and inactivates the active site of the enzyme. Resolving this presents an opportunity for enhancing the silver deposition. It should be noted that on other important POC diagnostic parameters, e.g., cost and portability, MO-BEAM already provides a significant advantage over ELISAs, even when used without its key feature of multiplexing. We summarize the comparison of platforms in Table 1.

**Table 1:** Comparison of Assay Characteristics between ELISA, LFA, and Our Portable Platform

method	sample volume (μL)	equipment cost	cost per test	time (min)	sensitivity (LOD, pM)	multiplexed	portable
ELISA	50	$20 K	$34–$60 [37]	120–180	0.26	No	No
LFA	10	0	$7–$10	15	34.72	No	Yes
MO-BEAM	3	<$100	$2	150	1.00	Yes	Yes

## Conclusions

We present a cost-effective and portable smartphone-based biosensing platform for multiplexed detection of biomarkers from small volumes of clinical samples. We term this as **M**ultiplexed **O**ptical **B**ioassay using **E**nzym**a**tic **M**etallization or **MO-BEAM**. This was developed by the integration of a simple detection technique based on enzymatically amplified silver metallization and a cellphone-based computer vision application for imaging and quantification of silver darkness related to the biomarker concentration. We demonstrated the use of MO-BEAM for the quantitative picomolar detection of SARS-CoV-2 antigen-specific antibodies from COVID-19 patient serum. We also used it to detect unique multiplexed viral antigen-specific antibody fingerprints from COVID-19 convalescent patient serum and uninfected vaccine recipients. This platform has the potential for being adapted for multiplexed detection of other disease biomarkers including antigens, proteins, DNA/RNA, viruses, bacteria, or even whole human or other mammalian cells. In addition, localized deposition property of the silver metallization provides the potential for the fabrication of massive multiplexed detection of larger numbers of biomarkers from a single droplet of sample on a miniaturized platform.

## Supporting Information Available

Stability testing (Figure S1); additional specificity testing (Figure S2); IgM response (Figure S3); serum ELISA results (Figure S4); monoclonal antibody ELISA results (Figure S5); comparison of MO-BEAM with ImageJ (Figure S6); and additional multiplexed detection results (Figure S7) (PDF)
