# Supplementary material for: Inexpensive High-Throughput Multiplexed Biomarker Detection Using Enzymatic Metallization with Cellphone-Based Computer Vision
Source: ACS Sens. 2023 Feb 8;8(2):534–42. doi: 10.1021/acssensors.2c01429 (PMC9972466; doi:10.1021/acssensors.2c01429)
Supplement: Supplementary file 1 — se2c01429_si_001.pdf [file se2c01429_si_001.pdf]

Inexpensive High-Throughput Multiplexed Biomarker Detection Using Enzymatic Metallization With Cellphone-Based Computer Vision

Neda Rafat‡, Lee Brewer‡, Nabojeet Das, Dhruti J. Trivedi, Balazs K. Kaszala, Aniruddh Sarkar*

Wallace H. Coulter Department of Biomedical Engineering, Georgia Institute of Technology, Atlanta GA 30332

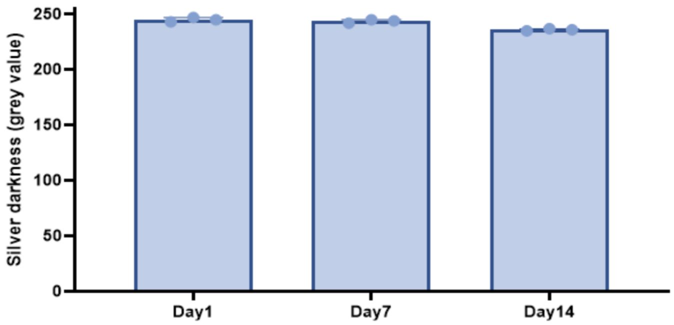

Figure S1. Testing stability of antigen immobilization on PLL-coated glass slide. An immunoassay was performed with Spike (S) antigen immobilized using 1µg/ml of human anti-Spike IgG monoclonal antibody (n=3).

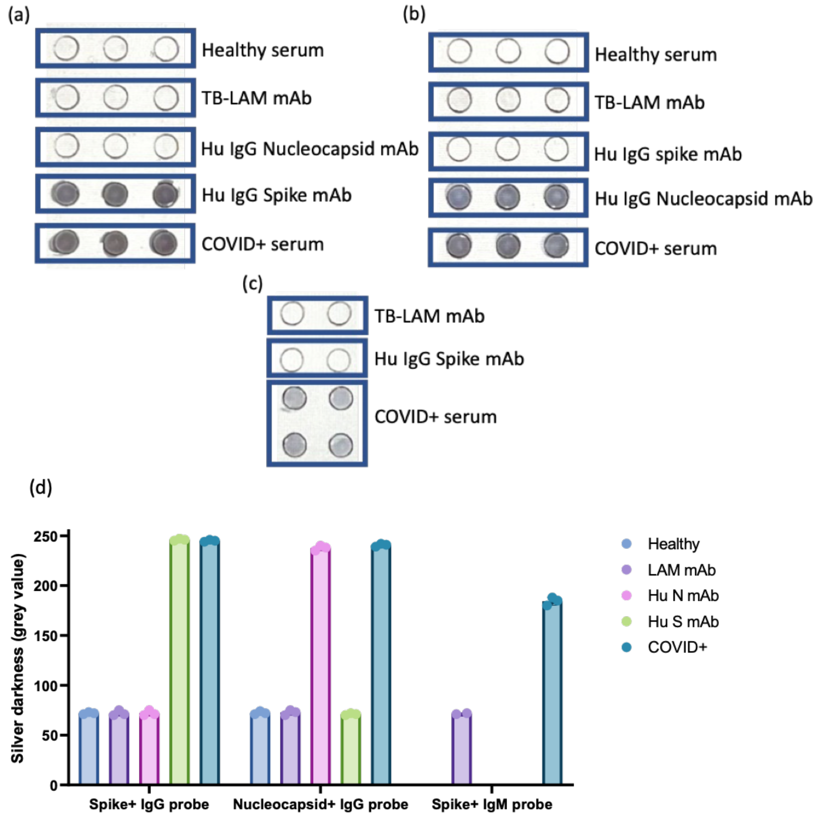

Figure S2. Additional testing of specificity. (a) Immobilized S antigen only react with samples containing antibodies against it. A probe against human IgG is used to detect the human IgG antibodies against S. (b) Immobilized nucleocapsid (N) antigen only react with samples containing antibodies against it. A probe against human IgG is used to detect the human IgG antibodies against N. (c) Immobilized S antigen on wells only react with samples containing antibodies against it. A probe against human IgM is used to detect the human IgM antibodies against spike. No cross reactivity is observed with Hu IgG spike mAb. (d) Quantification of additional specificity assays.

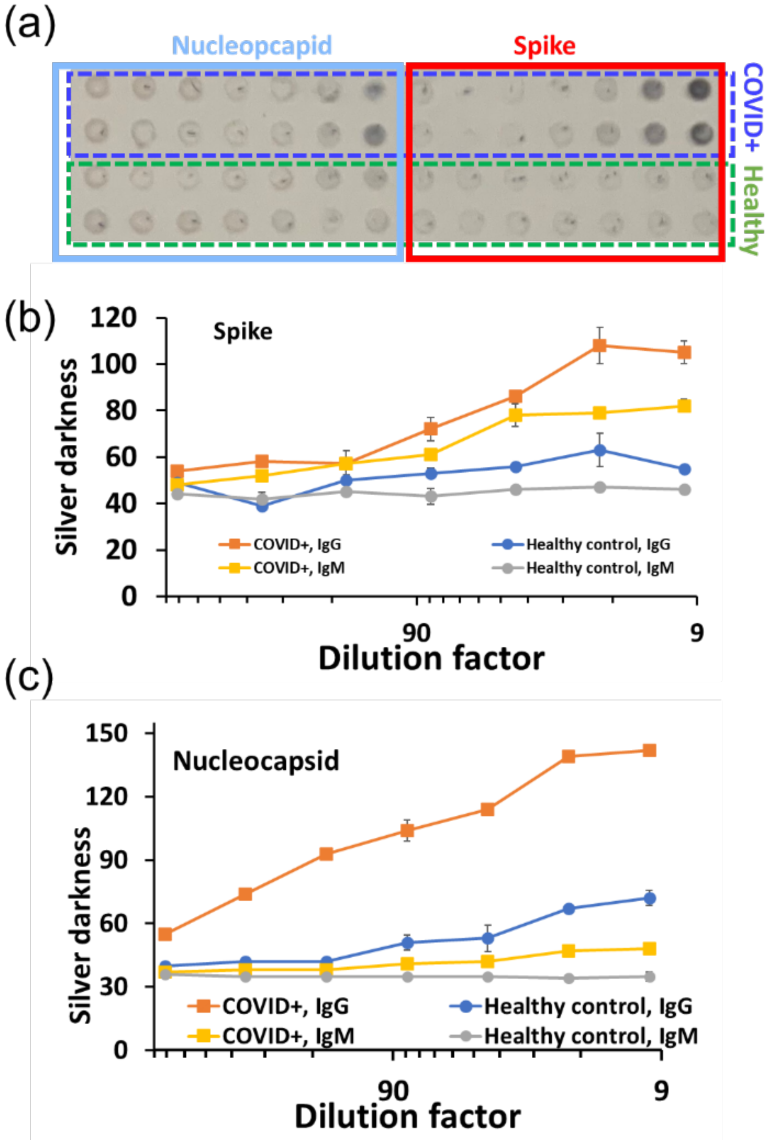

Figure S3. (a) Silver metallization deposited in response to human IgM COVID-19 antibodies. Quantification of silver darkness via the cellphone app for detection of anti-human IgG/IgM against (b) S or (c) N antigens

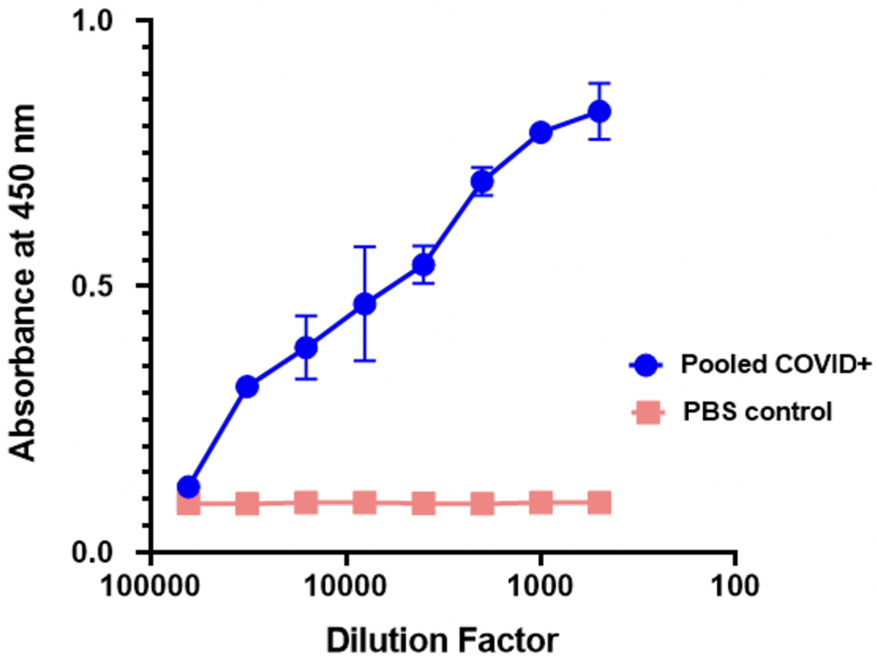

Figure S4. ELISA for serial dilution of COVID+ pooled serum sample and buffer (1XPBS) control.

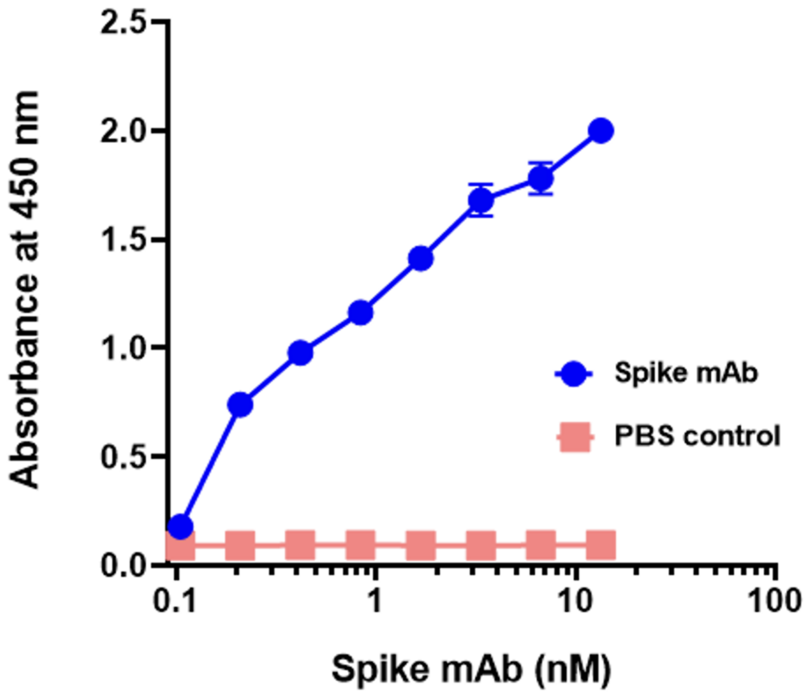

Figure S5. ELISA for serial dilution of anti-S human IgG monoclonal antibody and buffer (1XPBS) control.

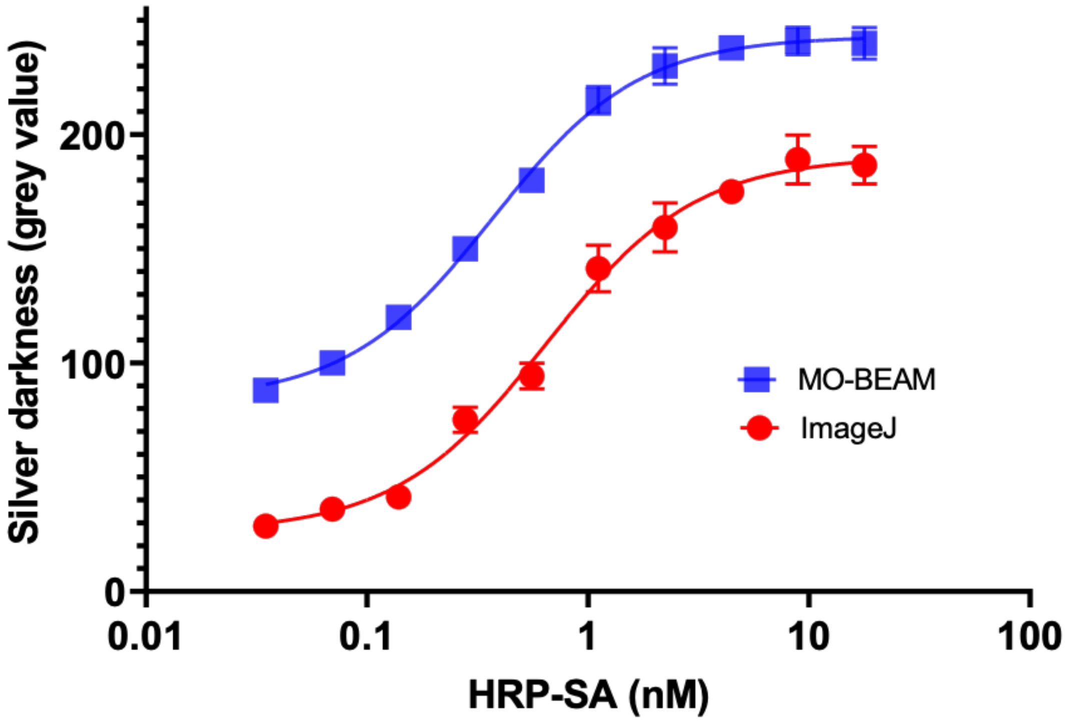

Figure S6. Comparison of assay quantification using ImageJ and optimized cell phone app.

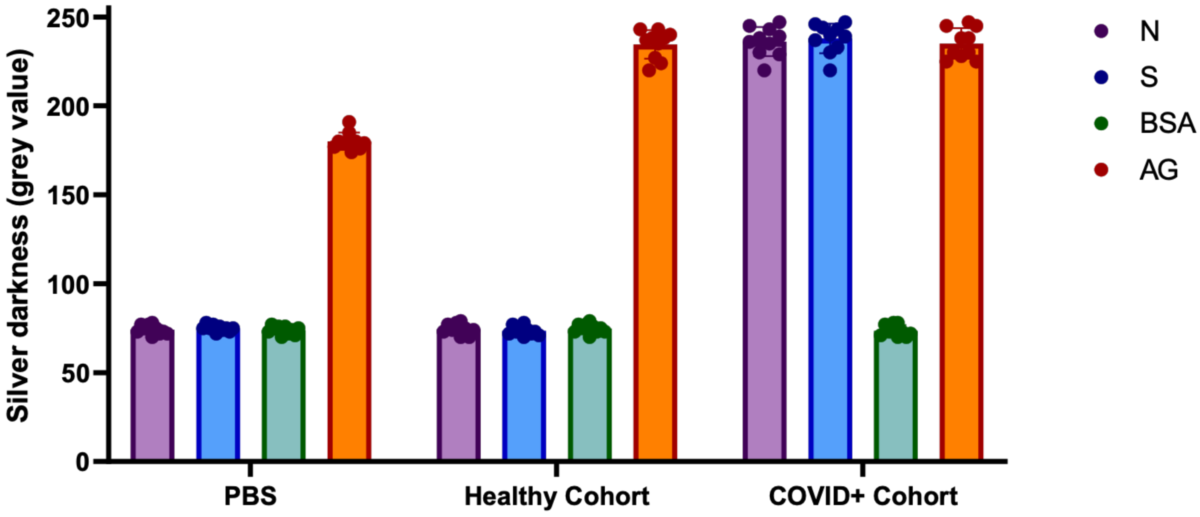

Figure S7. Multiplexed antibody detection of individual COVID+ patients (n=10), healthy controls (n=10) and buffer controls (1XPBS).
